# XCAVATOR: accurate detection and genotyping of copy number variants from second and third generation whole-genome sequencing experiments

**DOI:** 10.1186/s12864-017-4137-0

**Published:** 2017-09-21

**Authors:** Alberto Magi, Tommaso Pippucci, Carlo Sidore

**Affiliations:** 1 Department of Experimental and Clinical Medicine, University of Florence, Largo Brambilla 3, Florence, 50134 Italy; 2 Medical Genetics Unit, Department of Medical and Surgical Sciences, Polyclinic Sant’Orsola-Malpighi, University of Bologna, Via Zamboni 33, Bologna, 40126 Italy; 3 Istituto di Ricerca Genetica e Biomedica (IRGB), Cittadella Universitaria di Cagliari, Monserrato, 09042 Italy

**Keywords:** CNVs, HMM, Whole-Genome Sequencing

## Abstract

**Background:**

We developed a novel software package, XCAVATOR, for the identification of genomic regions involved in copy number variants/alterations (CNVs/CNAs) from short and long reads whole-genome sequencing experiments.

**Results:**

By using simulated and real datasets we showed that our tool, based on read count approach, is capable to predict the boundaries and the absolute number of DNA copies CNVs/CNAs with high resolutions. To demonstrate the power of our software we applied it to the analysis Illumina and Pacific Bioscencies data and we compared its performance to other ten state of the art tools.

**Conclusion:**

All the analyses we performed demonstrate that XCAVATOR is capable to detect germline and somatic CNVs/CNAs outperforming all the other tools we compared. XCAVATOR is freely available at http://sourceforge.net/projects/xcavator/.

**Electronic supplementary material:**

The online version of this article (doi:10.1186/s12864-017-4137-0) contains supplementary material, which is available to authorized users.

## Background

Copy number variants (CNVs) are operationally defined as 50 bp or larger DNA segments [1] that can be present at a variable copy number in comparison with a reference genome. CNVs have been demonstrated to be one of the main sources of genomic variation in humans [2–6] and have been shown to participate to phenotypic variation and adaptation by disrupting genes and altering gene dosage. Some CNVs can be apparently benign polymorphisms present at variable frequency in the population or confer susceptibility to various diseases including cancer, HIV acquisition and progression, cardiovascular, autoimmune, Alzheimer and Parkinson diseases [7].

Moreover, somatic copy number alterations (sCNAs) are common in cancer and the analysis of cancer genomes led to the identification of recurrent sCNAs in specific cancer types [8], and in some instances, of cancer causing-genes which are targets for tailored therapeutic approaches [9].

The last decade has seen the emergence of several second generation sequencing (SGS) platforms [10] that - by simultaneously generating billions of short DNA fragments (reads) - have revolutionized our ability to study genome variation and complexity. The advent of these technologies, together with the development of novel computational approaches, have transformed biological and biomedical research allowing the development of large-scale re-sequencing projects, such as the 1000 Genomes Project (1000GP) [11] or The Cancer Genome Atlas (TCGA, www.cancergenome.nih.gov), and opening a new era for personal genomics [12, 13]. The cost of SGS decreased so steeply that hundreds or even thousands of whole genomes can be now sequenced at affordable price. Whole genome sequencing (WGS) enables identification of point mutations and structural variations (SVs) of any size, ranging from small insertions/deletions to large CNVs, with unprecedented accuracy in determining position and orientation.

From a computational point of view, there are four main approaches for detecting SVs with SGS data that include read pair (RP), split read (SR), assembly methods (AS) and read count/depth of coverage (RC/DOC). RP methods identify insertions and deletions by comparing the distance between mapped paired reads to the average insert size of the genomic library. Although this method is able to identify deletions ≤1 kb with high sensitivity, it does not allow for the discovery of insertions larger than the average insert size of the library and of the exact borders of SVs in complex genomic regions rich in segmental duplication.

On the other hand, SR algorithms allow to detect deletions and small insertions by directly analyzing the mapping information and how high-throughput sequencing reads are aligned to the reference genome: a continuous stretch of gaps in the reads indicates a deletion while in the reference indicates an insertion. SR can detect, at least in theory, deletions without size limitation, while it can not detect insertions larger than read length, because the insertion can not be contained in a single read. The AS approach consists in a reference free, in silico, reconstruction of an entire genome consensus sequence from a collection of reads and its comparison with the reference genome by using softwares such as MUMmer [14], Mugsy [15], and Mauve [16] to discover structural differences. However, owing to the short size of the reads generated by SGS technologies, assembly algorithms have been demonstrated to collapse in highly repeated and highly duplicated genomic regions [1], and although showing a lot of potential, their application as routine methods still needs further efforts, for both computational and technological developments.

The most recent approach for the detection of SVs is RC that is based on the assumption that the sequencing process is uniform and consequently the number of reads mapping to a region (the total coverage of a region) is expected to be proportional to the number of times the region appears in the DNA sample [17]. Following this idea, the absolute number of DNA copy of any genomic region can be inferred by counting the number reads/bases aligned to that particular region.

Although all the aforementioned approaches are capable to detect SVs with high accuracy, it has been shown that each method detects events with specific structural characteristics. A large fraction of events detected with RC approaches overlaps with annotated segmental duplications, while RP-specific events show a greater enrichment of simple repeat. RP and SR methods show the greatest extent of overlap [18]. RC and SR are the most discordant approaches, with fewer than 20% overlapping SVs detections, with the main differences primarily found in duplication- and repeat-rich regions.

At present, several tools have been introduced in literature for detecting SVs from WGS data and they include RC methods (CNV-seq [19], FREEC [20], CNANorm [21], HMMCopy, BICSeq [22], CNVnator [23], RDXplorer [24]), RP approaches (BreakDancer [25], PEMer [26], VariationHunter [27]), SR (Pindel [28], SVseq2 [29]) and also combined approaches that take advantage of the unique features of multiple tools (Genome Strip [30], Delly [31]). FREEC, CNANorm, HMMCopy and BICSeq were properly devised for the identification of somatic copy number variants by using pairs of matched tumor/normal samples, while other methods, such as RDXplorer, BreakDancer, CNVnator and PEMer are capable to study only germline CNVs. Moreover, all these tools are able to classify each genomic region with a three states classification scheme (deletion, normal, amplification) that is not capable to discriminate between two- and single-copy deletions and between three- and multiple-copies amplifications, thus limiting the use of sequencing data in the prediction of the exact number of DNA copies.

In order to overcome the limits of currently available methods, thanks to our experience in CNVs detection methods [17, 32–34], we developed a novel tool based on RC approach, XCAVATOR, that is capable to discover genomic regions involved in CNVs from WGS data. To this end we first studied the statistical properties and biases of RC data distribution, we developed a two-step procedure to mitigate the effect of these source of biases and we evaluated the capability of normalized RC to identify and predict the absolute number of DNA copies of deleted and duplicated genomic regions. As a further step, by using synthetic simulations, we demonstrated that our shifting level model (SLM) algorithm is capable to detect the boundaries of deletions and duplications from RC genomic profiles outperforming the state of the art circular binary segmentation (CBS) approach. Finally we combined our normalization and segmentation approach with a calling procedure for the classification of each genomic region into five discrete copy number states (double deletion, single deletion, normal, duplication and multiple copy duplication) and we packaged them in the XCAVATOR tool.

To demonstrate the accuracy of our tool we applied it to the analysis of three different WGS datasets generated with second and third generation sequencing (TGS) technologies (929 WGS from the 1000GP, the TCGA benchmark dataset 4 and a genome sequenced with Pacific Bioscience long reads) and we compared its performance to other 10 state of the art tools for the identification of CNVs or sCNAs from WGS data. All the analyses we performed demonstrate that our computational pipeline is capable to detect germline CNVs and sCNAs in data from SGS and TGS experiments outperforming all the other tools we compared.

## Results

### RC bias distribution and normalization

The RC approach is based on the simple idea that during the sequencing process each read is randomly and independently sequenced from any location of the genome. Under this assumption the number of reads mapping to any region of the reference genome follows a Poisson distribution and is proportional to the number of times the region appears in the DNA sample: a genomic region that has been deleted (duplicated) will have less (more) reads mapping to it than a region not deleted (duplicated). Following this assumption, the copy number of any genomic region can be estimated by counting the number of reads (read count) aligned to consecutive and non overlapping windows of the genome.

In order to evaluate the capability of RC data to predict the copy number state of genomic regions involved in deletions and duplications, we studied the statistical properties and biases of RC distribution by using the high coverage genomes sequenced by Eberle et al. with the Illumina HiSeq platform (see “Methods” section for more details). To better understand the properties of RC distribution, the original 50x WGS experiments were downsampled to simulate sequencing coverages from 5x to 50x (see “Methods” section) and RC were calculated for four different window sizes (100 bp, 200 bp, 500 bp and 1000 bp).

**Fig. 1:**
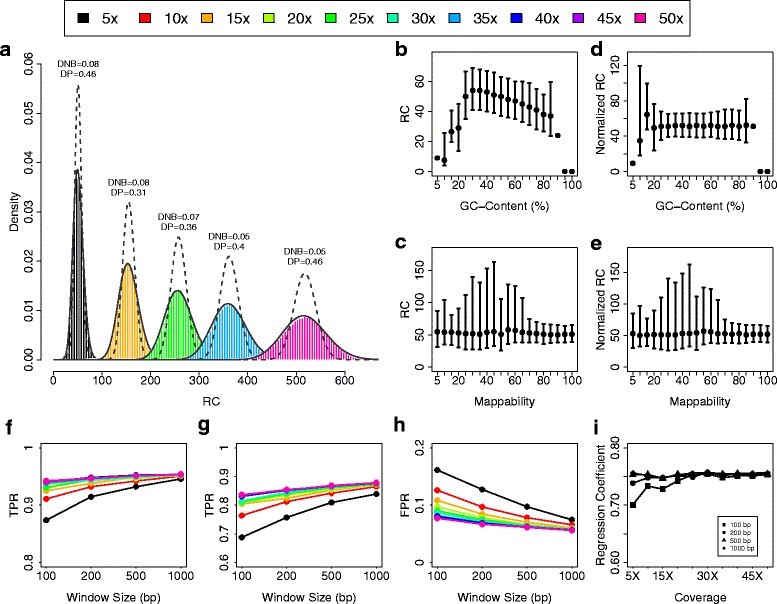
RC data distribution, biases normalization and CNV prediction capabilities. Panel **a** shows the histograms of RC data from Illumina Platinum WGS experiments for different sequencing coverages with the superimposed Poisson (dashed lines) and negative binomial (solid lines) distributions. The distance between RC and Poisson (DP) and negative binomial (DNB) distributions are calculated with the Kolmogorov-Smirnov statistic D. Panels **b** and **d** report the correlation between RC data and GC content % and mappability. Panels **c** and **e** show the effect of median normalization approach on GC content % and mappability. Panels **f**, **g** and **h** summarize the capability of RC data to detect deletions (**f**), duplications (**g**) and false positive events (**h**) as a function of sequencing coverage and window size. The TPR of panels **f** and **g** are the proportion of correctly predicted simulated deletions and duplications respectively. The FPR of panel h is the proportion of 2 copies regions predicted as deletions or duplications. Panel **i** show the association (regression coefficient) between real and predicted number of copies as a function of sequencing coverage and window size

The results summarized in Fig. 1a show that RC data are better modeled by means of a negative binomial distribution than a Poisson distribution (the Kolmogorov-Smirnov statistic DNB is smaller than DP for all sequencing coverages). In accordance with the results reported in [17, 24], we found that RC distribution exhibits an index of dispersion (ratio between variance and mean) largely greater than one and this overdispersion can be ascribed to two main sources that are local GC content and mappability, defined as the inverse of the number of times that a sequence originating from any position in the reference genome maps to the genome itself (Fig. 1b-c).

The correlation between RC and GC content has been previously reported in several papers for SGS data and is mainly due to the amplification step of the sequencing process. On the other hand, mappability bias is generated by ambiguous read alignments in genomic regions with many repetitive elements. By analyzing Illumina, 454 and SoLID reads we observed that RC is maximum for values of GC content between 35% and 60% while it decreases at both extremes [17]. In the same paper, we also found that RC distribution for highly mappable regions is closer to Poissonian than genomic regions with low mappability.

In order to mitigate the effect of these biases and make data comparable within and between samples, RCs need to be normalized. In [17] we demonstrated that the GC-content and mappability effects can be minimized by using the median normalization approach (see “Methods” section). The results reported in Fig. 1d-e and Additional file 1: Figures S1 and S2 demonstrate that median normalization approaches are able to mitigate the effect of the two sources of bias, equalizing the mean level of each bin to the same master mean.

As a further step, in order to understand the capability of the RC data to discriminate altered and normal genomic regions of different size, we generated synthetic deletions, duplications and normal copy regions made of different number of windows by sampling RCs from genomic regions previously predicted as deleted, duplicated and normal by McCarroll et al. [35] (see “Methods” section). To deeply understand the resolution limits of the RC approach, the simulations were performed for different sequencing coverages (from 5x to 50x) and window sizes (100 bp, 200 bp, 500 bp and 1000 bp). As expected, the larger the coverage and window size and the higher the capability to predict deletions and duplications made of small number of RC windows and the smaller the number of false positive events (Fig. 1f-h and Additional file 1: Figure S3). This is due to the fact that higher coverages and larger windows reduce the signal to noise ratio of RC distributions.

Finally, to test the effect of window size and sequencing coverage on the capability of RC data to predict the absolute number of DNA copies of genomic regions, we studied the RC distribution of genomic regions predicted to have copy number from 0 to 6. In particular, we performed regression analysis for estimating the relationships between two-copies normalized RC and the copy number of genomic regions previously genotyped by McCarroll et al. Also in this case, the larger the coverage and window size and the higher the correlation between real and predicted copy number states (Fig. 1i and Additional file 1: Figure S4). Taken as a whole, these simulation analyses demonstrate that for high sequencing coverages (≥30*x*) the use of small window sizes (100/200 bp) gives high resolution (high true positive rate (TPR) and accurate copy number states prediction) with low increase of false positive rate (FPR), while for low sequencing coverages (≤10*x*) it is preferable to use larger window size (500/1000 bp) to obtain good TPR and FPR.

### RC signal analysis

Once the RCs have been corrected for local GC content and mappability and ordered for genomic position, the data that we obtain are noisy signals in which deletions or duplications are identified as decrease or increase of RC across multiple consecutive windows. Although the correction for GC and mappability substantially reduces the biases that affect RCs, they are still affected by noise caused by mapping errors and random fluctuations in genome coverage and, as demonstrated in previous section, strongly dependent on average sequencing coverage and the choice of window size. For these reasons, the detection of CNVs from RCs need very sophisticated algorithmic recipes that are capable to identify with high precision the boundaries (segmentation) of increased or decreased RC data.

In the last few years we developed a class of algorithms, based on shifting level models (SLM), that allow to segment genomic profiles generated by high throughput platforms (see “Methods” section). The first SLM algorithm [32] was developed for analyzing log _2_-ratio data from array-CGH, the multivariate version, JointSLM [33] was written for the joint segmentation of multiple RC signals, while the heterogeneous version, HSLM [34] was properly tailored for segmenting spatially sparse data from whole exome sequencing experiments.

RC signals from WGS experiments are obtained by dividing the genome into consecutive and non overlapping windows of the same size and for this reason, the best SLM approach for segmenting WGS RC signals is the classical homogeneous SLM algorithm [32]. Moreover, since our SLM method is based on gaussian distributions, normalized RC data need to be log-transformed to obtain signals that are as close as possible to a normal distribution (see Additional file 1: Figure S5).

To test the ability of the SLM algorithm to detect CNVs of different size from WGS signals as a function of window size and sequencing coverage, we performed an intensive simulation based on synthetic chromosomes generated from the RC data of the platinum genomes described in the previous section (see “Methods” section).

RC data were calculated for four different window length (100 bp, 200 bp, 500 bp and 1000 bp), corrected for GC-content and mappability bias, median normalized to two-copies and then log _2_-transformed. Each synthetic chromosome was made of 1000 log _2_-RC windows and contained one deletion or duplication placed at random position. The copy number states of each synthetic chromosome were simulated by exploiting the results obtained by McCarroll et al. [35]:

- normal copy regions were simulated by sampling (1000-N) log _2_-RC windows from genomic regions previously predicted as 2-copies by [35]
- deletions (duplications) were simulated by sampling N log _2_-RC windows from regions previously predicted as deleted (duplicated) by [35].

**Fig. 2:**
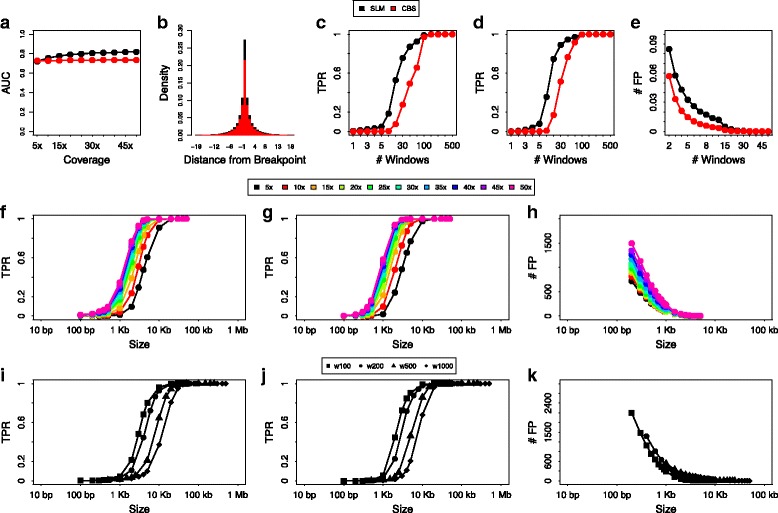
SLM and FastCall performance. SLM and FastCall performance. Panels **a-e** compare the performance of SLM and CBS algorithms on the analysis of synthetic chromosomes. Panel **a** reports the area under the ROC curve of SLM and CBS for different sequencing coverages, while panel **b** summarize the performance of SLM and CBS in the detection of the correct breakpoint position. On the x axis is reported the distance between the predicted and the correct position. On the y axis is reported the fraction of breakpoints predicted at a given distance from the correct position. Panels c-e show TPR (**c** for deletions, **d** for duplications) and # FP **e** for SLM and CBS as a function of number of windows. # FP of panel **e** is calculated as the average number of false positive events detected in all the synthetic chromosome we simulated. Panels f-k report the resolution of SLM+FastCall algorithms for different sequencing coverages (**f-h**) and different window size (**i-k**). Resolution results are reported for deletions (**f** and **i**), duplications (**g** and **j**) and false positive events (**h** and **k**). # FP in panels **h** and **k** is estimated as the total number of false positive events we can expect from the analysis of an entire human genome

For each window size and sequencing coverage, we performed simulations with N variable from 1 to 500 (see “Methods” section). As a first step, to assess the accuracy of SLM in detecting CNV boundaries we calculated the area under the receiver operating characteristic (AUC) curve and we compared its performance with the CBS algorithm that is the most widely used and cited algorithm for segmenting genomic profiles from aCGH and NGS experiments. Moreover, to assess the ability of our segmentation algorithm in correctly identifying the exact breakpoint of a CNV region, for each synthetic chromosome we calculated the distance (in windows) between the predicted and the correct breakpoint position and we compared its performance with CBS. The results of Fig. 2a-b and Additional file 1: Figures S6 and S7 demonstrate that our segmentation approach outperforms the CBS for all the alteration size we simulated and is capable to detect breakpoints with higher precision.

As a further step, in order to study the sensitivity and specificity of SLM and CBS in detecting altered segments made of different number of windows, we evaluated TPR and number of false positive events (# FP) as in [24, 33]: a detected segment is considered a true positive (TP) if there is a reciprocal overlap larger than or equal to 50% between the detected segment and the synthetic altered region, while it is considered a false positive (FP) if the reciprocal overlap with a synthetic altered region is smaller than 50%. Figure 2c-e and Additional file 1: Figure S8 show that, although SLM detects a slightly higher fraction of small FP events, it outperforms CBS in the detection of both deletions and duplications for all window sizes.

Genomic segments detected by SLM need to be classified into discrete copy number states. In [36] we introduced FastCall, an algorithm based on a mixture of truncated gaussian distribution that is able to classify segmented regions according to a five state classification scheme (2-copies deletion, 1-copy deletion, normal, 1-copy duplication and multiple-copies amplification). In order to evaluate the performance and resolution of SLM and FastCall methods in detecting CNVs of different size we applied them to the aforementioned synthetic chromosomes. Figure 2f-h and Additional file 1: Figure S9 show that for high sequencing coverage (≥30*x*) our approach is capable to detect CNVs as short as 5 kb (TPR ≥0.9), while for low coverages (≤10*x*) the resolution decreases to CNVs as short as 10 kb. However, although the adoption of small window size increases resolution, this is obtained at the expense of an increased number of FP events (Fig. 2i-k and Additional file 1: Figure S10). These results are in accordance with those obtained in previous section, and suggest to use small window sizes (100/200 bp) to increase resolution (in particular for high coverage data) and larger window size (500/1000 bp) to contain FPR (in particular for low coverage data).

Remarkably, all the synthetic analysis we performed in this section show that both CBS and SLM detect 1-copy regions with higher sensitivity than 3-copy regions and this result can be mainly ascribed to the fact that the variance of RC data is lower for deleted states (zero or one copy) and it proportionally increases with copy number values [24]: the larger is the variance and the smaller is the sensitivity of segmentation algorithms in detecting signal shifts.

### Population data analysis

To demonstrate the validity of our computational approach in population genomic studies, we used XCAVATOR to analyze 929 low coverage WGS experiments (from 4x to 15x) generated by the 1000GP consortium with the Illumina platform and comprising individuals from 14 different sub populations (see Materials and Methods for more details). To evaluate the performance of our tool, we compared its results with those obtained by other six state-of-the-art methods used in Phase 1 of the 1000GP for the detection of bi-allelic deletions and that include two RP algorithms (Pindel and BreakDancer), two RC approaches (CNVnator and RDXplorer) and two combined methods (Delly and GenomeStrip).

**Fig. 3:**
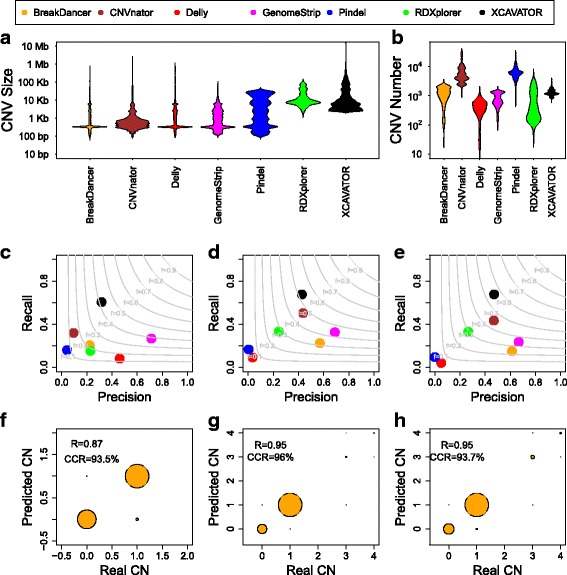
Summary of the results obtained by XCAVATOR on the 1000 Genomes Project samples. Panels **a** and **b** show the distribution of the size (**a**) and number (**b**) of CNVs detected by XCAVATOR and the other six state of the art tools. Panels **c**, **d** and **e** report precision and recall obtained by the seven tools in detecting CNVs previously identified by 1000GP pilot 1 (**c**), HapMap (**d**) and McCarroll (**e**). Light grey curves represent F-measure levels (harmonic mean of precision and recall). In panels **f**, **g** and **h** are reported the correlations between the absolute number of DNA copies inferred by SLM+FastCall and those previously estimated by 1000GP pilot 1 (**f**), HapMap (**g**) and McCarroll (**h**). R is the Pearson correlation coefficient and CCR is the correct classification rate, calculated as the proportion of genomic regions with the correct number of DNA copies

Since the great majority of 1000GP WGS experiments have coverage smaller than 10x, in order to maximize TPR and minimize FPR, according with the results obtained in “RC bias distribution and normalization” section, we decided to run our tool with a window size of 1000 bp. By using this window size, XCAVATOR detected CNVs with a size distribution that range from 1 kb to 10 Mb (Fig. 3a-b). Conversely, the size distribution of the SVs detected by five of the 1000GP methods (Delly, GenomeStrip, CNVnator, BreakDancer and Pindel) are mainly concentrated between 100 bp and 10 kb, with the exception of RDXplorer that detect CNVs with length similar to our tool.

Recently, Sudmant et al., by analyzing the low coverage WGS data of 2,504 unrelated individuals from 1000GP phase 3, identified around 69,000 SVs that range from 100 bp to 1 Mb and with a significant fraction below 10 kb. To obtain this high resolution SVs map, they used a very complex computational and experimental strategy based on the combination of nine state-of-the-art tools (BreakDancer, Delly, VariationHunter, CNVnator, Read-Depth, GenomeStrip, Pindel, MELT and Dinumt) and on validations with other techniques that include PacBio, Nanopore and Complete Genomics sequences and Microarray technologies. The results obtained with our tool (Fig. 3a) demonstrate that XCAVATOR is capable to explore a large spectrum of SV events (from 1 kb to 10 Mb) with respect to other state of the art methods. Moreover, while XCAVATOR detected a comparable number of CNV events (from 800 to 2000 with an average number of 1000) across analyzed sample, the number of SVs per individual identified by the six 1000GP approaches is very variable (Fig. 3b), with CNVnator and Pindel that detected more than 20,000 events per sample.

To study sensitivity and specificity of all these methods, we compared these seven sets of calls with the CNVs previously identified by McCarroll et al. [35], the HapMap [37] and the 1000GP Pilot 1 [18] projects in the same samples. The CNV regions of HapMap and McCarroll datasets were genotyped by using SNP-array technologies, while the 1000GP pilot CNVs were discovered by using several computational approaches (based on RD, PEM and SR) applied on the low-coverage WGS experiments of 179 individuals (see “Methods” section for more details).

The technological and computational nature of the events present in the three validation datasets allowed us to deeply examinate the detection power of our approach and the other six tools in the identification of different classes of structural variants. To measure the performance of the seven tools, for each of the three reference sets, we calculated precision as the ratio between the number of correctly detected events (the intersection between the tool calls and the validation set calls) and the total number of events detected by a tool. The recall was calculated as the ratio between the number of correctly detected events and the total number of events in the validation set. An event was considered correctly detected if we found a reciprocal overlap larger than or equal to 50% with a CNV of the validation set. The 50% reciprocal overlap criterion has been previously used is several papers for evaluating the performance of algorithms for CNVs identification [23, 27, 29].

Since the capability of detecting regions involved in CNVs is highly influenced by their size, precision-recall analysis was performed taking into account all event size (Fig. 3c-e) and distinguishing three size classes (Additional file 1: Figure S11): Small (*s* *i* *z* *e*≤20*k* *b*), Medium (20*k* *b*<*s* *i* *z* *e*≤100*k* *b*) and Large (*s* *i* *z* *e*>100*k* *b*). Moreover, since the structural variants reported in the 1000GP Phase 1 for the six state of the art approaches contain only deletions, we calculated precision and recall taking into account only heterozygous and homozygous deletions also for XCAVATOR.

The results reported in Fig. 3c-e and Additional file 1: Figure S11 clearly show that our approach outperforms the other six tools in terms of trade off between precision and recall both precision and recall for all the three validation dataset in the three size classes. Globally, CNVnator and XCAVATOR obtained the best F-measure in the identification of large and medium events, while small variants were best identified by XCAVATOR and GenomeStrip. RDXplorer obtained good results in the detection of medium size deletions, but completely failed the identification of large events. GenomeStrip best performed in small deletions identification, while Pindel, Delly and BreakDancer obtained poor results in all the three size classes.

**Fig. 4:**
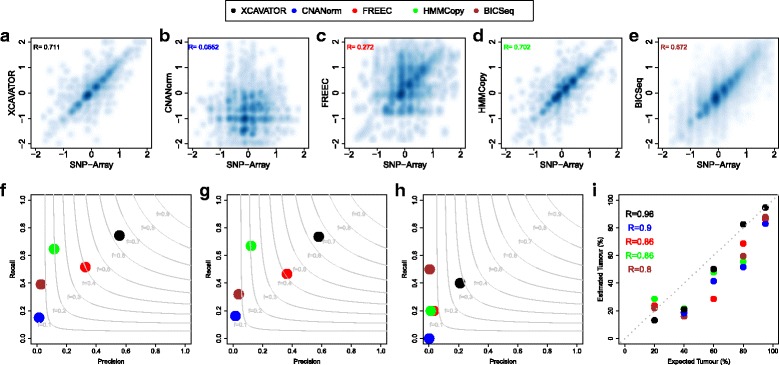
TCGA benchmark 4 results. Panels (a-e) show the density plots of the the relationship between the *l* *o* *g*_2_−*r* *a* *t* *i* *o* median values obtained from WGS and SNP-array for genomic segments detected by XCAVATOR (**a**), CNANorm (**b**), FREEC (**c**), HMMCopy (**d**) and BICSeq (**e**). Panels **f-h** report precision and recall obtained by the five tools in detecting all (**f**), small (**g**) and large (**h**) sCNAs identified by SNP-array data. Light grey curves represent F-measure levels (harmonic mean of precision and recall). In panel **i** are reported the correlation between real contamination and those estimated by THEtA using sCNAs detected by the five tools. R is the Pearson correlation coefficient

Panels c-e of Fig. 4 also show that XCAVATOR and CNVnator obtained high recall rate in almost all the validation datasets, while GenomeStrip and BreakDancer have high precision. As expected [24], the three RC approaches (XCAVATOR, CNVnator and RDXplorer) best performed in the recognition of array-based events, while the multiple approach (GenomeStrip) obtained good results in the 1000GP dataset. Although most of the 1000GP events were called by the six tools used in this comparison analysis, our approach is capable to obtain very good results in the identification of the deletions of this validation dataset, demonstrating its uniqueness in the analysis of structural variants from WGS data.

As a final step, in order to evaluate the capability of our tool to infer the absolute number of DNA copies, we studied the correlation between the copy number predicted by XCAVATOR and those present in the three validation datasets (see “Methods” section for more details). The results reported in Fig. 3f-h and Additional file 1: Figure S12 show that our tool is capable to correctly infer the exact number of DNA copies of genomic regions of any size class.

### Somatic copy number variants detection

sCNAs are acquired by cancer genomes during the carcinogenesis and the duplication of oncogenes or the deletion of tumor suppressor genes can have pathogenic effect. In particular, genomic regions involved in recurrent sCNAs (shared by several cancer samples) have high probability to contain cancer genes and several cancer-related genes have been identified thanks to sCNAs detection. Moreover, patterns of sCNAs can divide cancer types into different subgroups with different prognostics and treatment responses.

To show the power of our tool to detect sCNAs we used XCAVATOR to analyze the TCGA Benchmark 4 dataset that comprises WGS experiments obtained from two pairs of cell lines derived from grade 3 breast ductal carcinomas and the corresponding patient matched normal samples derived from blood (see “Methods” section). To measure the performance of our method we compared its results with those obtained by other four state of the art tools (FREEC, CNANorm, HMMCopy and BIC-Seq) using a 250K SNP-array dataset as gold standard (see “Methods” section). Since the average physical distance between consecutive probes in 250K SNP-array is around 1.2 kb, in order to obtain comparable CNA profiles, XCAVATOR, FREEC, CNANorm, HMMCopy and BICSeq were run with default parameter setting using a windows size of 1000 bp.

As a first step, to evaluate the accuracy of the genomic profiles generated by each method, we compared the log _2_-ratio median values obtained from WGS and SNP-array data of each segmented region detected by XCAVATOR and the other four state of the art tools, considering small (≤20*k* *b*) and large (≥20*k* *b*) genomic segments separately. The results reported in Fig. 4a-e and Additional file 1: Figure S13 show that the computational pipeline at the base of our tool is capable to better predict the copy number states of cancer genomes obtaining the best results in terms of correlation coefficients for both large and small genomic segments followed by HMMCopy, BICSeq, FREEC and CNANorm. As a further step, to test the capability of XCAVATOR and the other four tools to correctly detect alterations of different size, we calculated precision and recall as in previous section (with a 50% reciprocal overlap criterion) by using sCNAs detected with SNP-array as reference set (see “Methods” section) and we found that our tool (Fig. 4f-h) outperforms the other methods in the detection of both large (≥20*k* *b*) and small (≤20*k* *b*) CNAs.

Due to the contamination and the subclonal nature of cancer samples, a given sCNA can only be found in a fraction of cancer cells generating genomic profiles with weak shifts that reflect the allelic fraction of somatic events. The signal shifts generated by sCNAs in RC profiles can be exploited to estimate tumor contamination and characterize the subclonal populations present in a cancer sample. In order to demonstrate the power of XCAVATOR to correctly detect weak signals generated by low frequency sCNA, we exploited the WGS experiments with varying levels of normal contamination and tumor samples simulated in the TCGA Benchmark 4 dataset (see “Methods” section). In particular, we combined the copy number profiles generated by the five methods with the Tumor Heterogeneity Analysis (THetA) tool [38]. THetA is a computational approach that infers the most likely collection of genomes and their proportions starting from copy number aberrations profiles detected by high-throughput DNA sequencing data. The results of Fig. 4i show that THetA obtain the best estimation results by using the CNA profiles generated with XCAVATOR, demonstrating the capability of our tool to manage even low frequency events with high precision.

### Third generation sequencing

As reported in previous sections, the absolute number of DNA copies of any genomic region is inferred, in the RC approach, by counting the number of reads that map to consecutive and non overlapping windows of the reference genome. Each read is uniquely assigned to a window when its first mapping position falls into the window interval. When window size is larger than read length, RC is a good approximation of the total sequencing throughput mapping to a region, while, conversely, when read length is much larger than window size, counting reads can generate windows with low RCs, even at high sequencing coverages. In this situation, it is preferable to measure the total sequencing throughput of a region by using the average sequencing depth of coverage (DOC) of each window.

The last few years have seen the emergence of a new generation of sequencing platforms based on single-molecule real-time (Pacific Bioscience, PacBio) [39] and nanopore sequencing (MinION) [40], that interrogate single molecule of DNA and are capable to produce sequences in the order of tens of Kb in size. In [41] we demonstrated that both MinION and PacBio reads can be used to detect genomic regions involved in CNVs by means of the DOC approach with an accuracy comparable to that of Illumina data.

To demonstrate that our computational pipeline is capable to analyze TGS data, we studied the statistical properties of the DOC distribution of SMRT Pacific Bioscience sequences generated by the Genome in a Bottle Consortium (GIAB) for the NA12878 sample (see “Methods” section for more details).

**Fig. 5:**
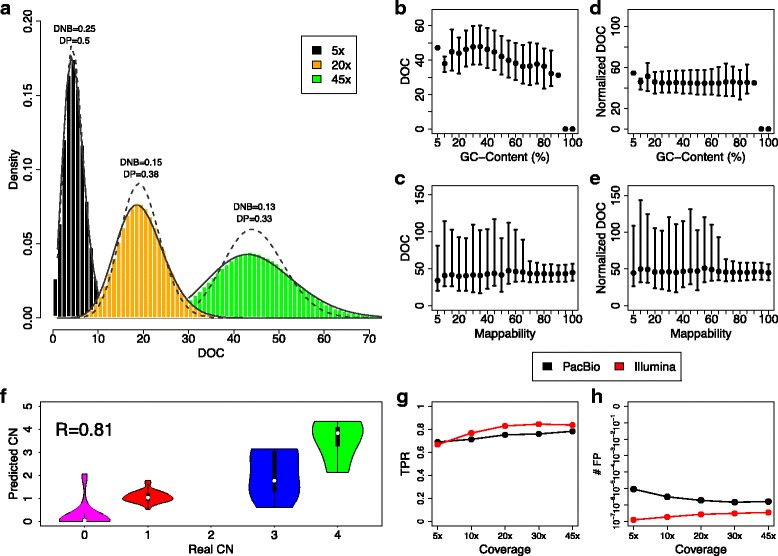
XCAVATOR results on the TGS PacBio data. Panel **a** shows the histograms of DOC data fromf the PacBio WGS experiments for different sequencing coverages with the superimposed Poisson (dashed lines) and negative binomial (solid lines) distributions. The distance between RC and Poisson (DP) and negative binomial (DNB) distributions are calculated with the Kolmogorov-Smirnov statistic D. Panels **b** and **d** report the correlation between DOC data and GC content % and mappability. Panels **c** and **e** show the effect of median normalization approach on GC content % and mappability. The violin plots of panel **f** show the correlation between absolute number of DNA copies obtained by DOC signals and those previously estimated by McCarroll et al. Panels **g** and **h** summarize the TPR and number of FP detected by XCAVATOR with PacBio and Illumina data

In order to better understand the statistical properties of DOC data as a function of sequencing coverage, we downsampled the 45x experiment to simulate 5x, 10x, 20x and 30x experiments. As previously reported in [41], PacBio DOC data are distributed as a negative binomial distribution and classical GC-content and mappability biases can be mitigated with median normalization approaches implemented in XCAVATOR (Fig. 5a-e). To study the capability of PacBio data to predict the exact number of DNA copies of genomic regions involved in CNVs, we calculated the average value of normalized DOC signal of regions previously predicted duplicated or deleted by [35]. The results reported in Fig. 5f and Additional file 1: Figure S14 demonstrate that normalized DOC data are highly correlated with the CNV states of any genomic region.

As a further step, to evaluate sensitivity and specificity of XCAVATOR in the analysis of DOC signals from long reds data (see Materials and Methods), we calculated TPR and FP as in “RC signal analysis” section and we compared the results with those obtained with Illumina SGS data (Fig. 5g-h and Additional file 1: Figure S15). Although the genomic profiles of PacBio data generate an high number of FP they give a sensitivity similar to Illumina profiles, demonstrating that our approach is capable to manage also this new generation of genomic profiles even at low sequencing coverages.

## Implementation

All the computational approaches described in this paper have been packaged in the XCAVATOR software tool. XCAVATOR is a collection of perl, bash, R and fortran codes and its computational architecture has been derived from the EXCAVATOR tool that we published in 2013 [34] for the detection of CNVs/sCNA from whole-exome sequencing data. Our tool takes as input WGS data as BAM files and gives as output plots reporting raw, normalized, segmented and called data and a list of detected CNVs in tab-delimited and VCF format.

The tool allows to analyze WGS data with three different experimental designs: “pooling”, “paired” and “nocontrol”. In “paired” mode each test sample is compared with its matched control and it is the best scheme to detect sCNA from pairs of tumor and matched normal samples. The “paired” mode has been used to analyze the TCGA Benchmark 4 dataset. In “nocontrol” mode the RCs of each test sample are normalized to two copies and this scheme is best suited for population genomics studies and has been used to analyze the 929 WGS experiments sequenced by 1000GP consortium. In “pooling” scheme, each test sample is compared to a pool control samples by summing the RC of each window across all the control samples. XCAVATOR can run on any unix system (desktop and servers) and allows the user to set the number of processor to analyze multiple samples in parallel. On a desktop computer with a 2.5 GHz cpu and 8 GB of ram, by using four cores, it takes four hours to perform the analysis of ten WGS samples sequenced at 60x. The XCAVATOR tool is freely available at https://sourceforge.net/projects/xcavator/.

## Conclusion

In the last few years, the cost of sequencing experiments has decreased so steeply that it is now possible to analyze large cohorts of whole genomes at affordable prices. In this scenario, the availability of software tools that are capable to handle the data generated with this experimental strategies is of fundamental importance. In this work, we present a novel software tool based on RC approach for the detection of CNV or sCNAs from WGS experiments generated by SGS or TGS platforms. We studied the statistical properties and biases of RC data as a function of sequencing coverage, we introduce a two step normalization procedure to mitigate the effects of these biases and we demonstrated that normalized RC data are capable to predict the exact number of DNA copies of genomic regions involved in CNVs. As a further step, to analyze the RC genomic profiles generated by WGS experiments we tested two algorithms originally devised for the analysis of array-CGH data, SLM and FastCall.

By using synthetic simulations we demonstrated that our segmentation SLM algorithm outperforms the state of the art CBS method in terms of both sensitivity and specificity and that the combination of SLM with the FastCall calling method is capable to detect CNVs as long as 10 kb with low coverage WSG experiments and as long as 1 kb with high coverage WGS data. The two median normalization methods and the SLM and FastCall algorithms were packaged in a novel software tool that we named XCAVATOR.

To demonstrate the power of our computational pipeline in the detection of genomic regions involved in CNVs or sCNAs, we used it for the analysis of three WGS datasets generated with two different sequencing technologies and we compared with other ten state of the art tools. To show the power of XCAVATOR in population genetics studies, we used it to analyze 929 low coverage WGS experiments sequenced by the 1000GP consortium and we compared the results with known CNVs from microarray and SGS studies. The results of these analyses show that our method obtains excellent performance in the detection of small, medium and large CNVs and that is capable to predict the exact number of DNA copies with high accuracy.

As a further step, we tested our tool in cancer genomic studies by using the TCGA Benchmark 4 dataset and comparing the results with those obtained with SNP-array data. Finally, we tested XCAVATOR in the analysis of WGS data generated by novel long reads sequencing platforms. The results obtained in this analysis demonstrated that our software tool is capable to handle this new generation of sequencing data detecting CNVs with a resolution comparable to that of SGS data. All the comparative analysis we performed in this work clearly demonstrate the versatility of our tool in the analysis of WGS data and its capability of overcoming the limits and drawbacks of currently available state of the art tools. Remarkably, while other state of the art methods allow to classify CNVs into three states (deletions, normal and duplications), XCAVATOR is capable to discriminate five copy number states, allowing to distinguish one-copy from two-copy deletions and one-copy duplications from multiple-copies amplifications.

## Methods

### RC bias distribution and normalization

Eberle at al. [42], by using the Illumina HiSeq system, generated the WGS data of 17 individuals of the CEPH pedigree (1463) at 50x sequencing coverage. Raw sequencing data in fastq format were downloaded from ENA at http://www.ebi.ac.uk/ena/data/view/PRJEB3381 and aligned to the human reference genome (hg19) with BWA mem using default parameter settings. Aligned reads were processed, sorted and filtered (discarding MQ ≤ 10) with SAMtools and PCR duplicates were removed with Picard MarkDuplicates (http://picard.sourceforge.net). To study the statistical properties of RC data, the original 50x experiments of NA12877, NA12878, NA12879 samples were downsampled at 5x, 10x, 15x, 20x, 25x, 30x, 35x, 40x and 45x with samtools view -s command.

To evaluate if RC data are distributed as a Poisson or negative binomial distribution we used the Kolmogorov-Smirnov statistics D that quantifies the distance between two empirical distribution function and the smaller is D the closer are the two distributions.

To mitigate the effect of GC content % and mappability biases we used a bias removal procedure based on the median normalization approach that we introduced in [17] and in [34]. For each GC percentages (0, 1, 2,..,100%) and each bin of mappability score (0, 0.1, 0.2,...,1) we calculated the deviation of RC or DOC from the window average and then corrected each RC according to the following formula:

1 $$ \overline{RC_{i}} = RC_{i}\cdot \frac {m} {m_{\mathrm{X}}},  $$

where *R* *C*_*i*_ are the window mean read counts of the *i*-th window, *m*_X_ is the median *RC* of all the windows that have the same X value (where X=[GC content, mappability score]) as the *i*-th window, and *m* is the overall median of all the windows.

For each window of the reference genome, GC content % was calculated with the nucBed command of bedtools [43] while the mappability score was estimated by using the gem-mappability module of the Genome Multitool (GEM) mapper [44]. To evaluate the capability of RC data to discriminate between normal and altered genomic regions, we used RCs normalized for GC content % and mappability and rescaled to copy number 2. Deletions, duplications and normal copy regions of different size were simulated by sampling N RC windows (N=1, 2, 3, 4, 5, 10, 20, 30, 40, 50, 100, 200, 300, 400 and 500) from genomic regions previously predicted as deleted, duplicated and 2 copies by McCarroll et al. [35]. Synthetic events were simulated for different window sizes (100 bp, 200 bp, 500 bp and 1000 bp). A simulated deletion was considered correctly predicted (TP) if the RC median value was ≤1.5. A simulated duplication was considered correctly predicted (TP) if the RC median value was ≥2.5. A simulated normal copy region was considered an FP if the RC median value was ≥2.5 or ≤1.5

### Segmentation and calling algorithms

In 2010 we introduced a powerful segmentation algorithm, based on SLM, for analyzing *l* *o* *g*_2_−*r* *a* *t* *i* *o* genomic profiles from array-CGH. SLMs [32] model noisy sequential processes with sudden shifts in the mean *x*=(*x*_1_,..,*x*_*i*_,..,*x*_*N*_) as the sum of two independent stochastic processes:

2 $$ x_{i}=m_{i}+\epsilon_{i},  $$

3 $$ m_{i}=(1-z_{i-1})\cdot m_{i-1}+z_{i-1}\cdot(\mu+\delta_{i}).  $$

where *m*_*i*_ is the unobserved mean level that follows a normal distribution with mean *μ* and variance $\sigma ^{2}_{\mu }\left (m_{i} \sim N\left (\mu, \sigma ^{2}_{\mu } \right)\right)$ and *ε*_*i*_ is a normally distributed white noise with variance $\sigma ^{2}_{\epsilon }\left (\epsilon _{i} \sim N\left (0, \sigma ^{2}_{\epsilon }\right), \text {Fig.~1a}\right)$.

The process *m*_*i*_ changes its value independently of *m*_*i*−1_ and is controlled by the process *z*_*i*_ : when *z*_*i*−1_=0, *m*_*i*_ is the same as *m*_*i*−1_ and when *z*_*i*−1_=1, *m*_*i*_ is incremented by the normal random variable $\delta _{i}\left (\delta _{i} \sim N\left (0,\sigma _{\mu }^{2}\right)\right)$. *z*_1_,*z*_2_,… are independent and identically distributed random variables taking the values 0,1 with probabilities *η*=*P* *r*(*z*_*i*_=1), 1−*η*=*P* *r*(*z*_*i*_=0).

It has been demonstrated that SLM is a special class of hidden markov models (HMM) and for this reason we can use classical HMM algorithms, such as Baum and Welch and Viterbi algorithms to estimate its parameters [32]. Once the RC or DOC signals have been segmented by SLM, each segmented region need to be classified into discrete copy number states. To this end we used the FastCall calling procedure, that is an ultra fast algorithm based on mixture of five truncated normal distributions and that is capable to classify each segmented genomic regions into five predefined copy number states: double loss, loss, neutral, gain and multiple gain. Our calling procedure is also able to take into account sample heterogeneity by means of a cellularity parameter c [34, 36].

### Population data analysis

In Phase 1, the 1000GP consortium analyzed the low-coverage WGS data of 1092 individuals to discover bi-allelic structural variants larger than 50 bp. To this end, they used several tools that include Delly, GenomeStrip, CNVnator, BreakDancer, RDXplorer and Pindel. We used XCAVATOR to analyze all the low-coverage WGS experiments sequenced with the Illumina platform (929 samples) and we compared its results with the CNV calls obtained by the 1000GP consortium with the six aforementioned methods. For all the six methods, CNV calls were downloaded at ftp://ftp-trace.ncbi.nih.gov/1000genomes/ftp/technical/working/20110531_phase1_sv_callsets/.

Concerning XCAVATOR analysis, RC data were first corrected for GC-content and mappability bias, median normalized to two-copies and then log _2_-transformed. In order to evaluate the performance of the seven methods, we calculated precision and recall by using the McCarroll et al., the HapMap and the 1000GP Pilot 1 datasets as gold standard. McCarroll et al. [35] developed a hybrid genotyping array (Affymetrix SNP 6.0) to interrogate 906,600 SNPs and copy number at 1.8 million genomic locations. All the experiments were performed in duplicates, using two different computational approaches for analyzing array data and using quantitative PCR to validate CNVs. Thanks to this strategy, they created a high resolution map of genomic regions involved in CNVs for 270 samples previously genotyped by the HapMap consortium. The HapMap consortium [37] identified 886 copy number polymorfisms by studying a set of 1184 individuals from 11 populations with a combination of two distinct SNP array platforms (Affimetrix 6.0 and Illumina 1M) and two algorithms for CNV detection (QuantiSNP27 and Birdseye5).

In phase Pilot 1, the 1000GP consortium [18] sequenced (with an average coverage of 3.63) the whole-genome of 179 samples that include 59 Yoruba individuals from Nigeria (YRI), 60 individuals of European ancestry from Utah (CEU), 30 of Han ancestry from Beijing (CHB), and 30 of Japanese ancestry from Tokyo (JPT). By using RP, SR, RC and AS approaches they detected 22,025 deletions most of which were mapped to nucleotide resolution. The McCarroll and HapMap datasets were downloaded from the Database of Genomic Variants (DGV, http://dgv.tcag.ca/dgv/app/home), while the 1000GP Pilot 1 dataset were downloaded from http://www.internationalgenome.org/.

### TCGA Benchmark 4

TCGA generated benchmark exercises for the comparative evaluation of of somatic mutation calls on single nucleotide variants (SNVs) and structural variants (SVs) under a variety of conditions designed to simulate the effects of tumor purity (i.e. normal contamination) by combining various fractions of tumor-derived and normal-derived cell line DNA.

The TCGA benchmark 4 dataset is made of high coverage WGS experiments derived from two pairs of grade 3 breast ductal carcinomas cell lines: HCC1143/HCC1143 BL and HCC1954/HCC1954 BL (where ’BL’ indicates the sample is the paired normal sample derived from blood).

To simulate varying levels of normal contamination in tumor samples there are two sets (one for HCC1143 and one for HCC1954) of six comparisons: normal 5% - Tumor 95%, normal 20% - Tumor 80%, normal 40% - Tumor 60%, normal 60% - Tumor 40%, normal 80% - Tumor 20%, normal 5% - Tumor 95%. To evaluate the performance of our tool in the detection of sCNAs we used it to analyze the two pairs of WGS experiments (HCC1143/HCC1143 BL and HCC1954/HCC1954 BL) and we compared its results to those obtained by FREEC, CNANorm, HMMCopy and BICSeq. XCAVATOR was run in “paired” mode, in which the log _2_-ratio between the normalized RCs from cancer and blood samples are segmented by SLM and called with FastCall.

To estimate the accuracy of the five tools we used Affymetrix GeneChip Human Mapping 250K data as gold standard (GEO code GSE34754). The normalized log _2_-ratio values between cancer and blood samples were segmented using the SLM segmentation algorithm followed by the FastCall calling procedure to classify all the segmented genomic regions into defined CN states.

### Pacific bioscience data

The Genome in a Bottle (GIAB) Consortium (https://github.com/genome-in-a-bottle) is creating diverse set of sequencing data for seven human genomes that include two Personal Genome Project trios, one of Ashkenazim Jewish ancestry and one of Asian ancestry and the NA12878 from the CEPH Utah Reference Collection [45].

The sequencing data come from 11 technologies: BioNano Genomics, Complete Genomics paired-end and LFR, Ion Proton exome, Oxford Nanopore, Pacific Biosciences, SOLiD, 10X Genomics GemCodeTM WGS, and Illumina paired-end, mate-pair, and synthetic long reads.

NA12878 sample was sequenced with the Pacific Biosciences Single Molecule Real-Time (SMRT) platform using the P6-C4 chemistry and obtaining a 45x total sequencing coverage. The bam files of PacBio reads mapped to the human reference genome (hg19) with BLASR (v1.3.2) were downloaded from ftp://ftp-trace.ncbi.nih.gov/giab/ftp/data/NA12878/NA12878_PacBio_MtSinai/. To simulate different sequencing coverage (5x, 10x, 20x, 30x), the original 45x bam file were downsampled with samtools view -s.

DOC data were first corrected for GC-content and mappability bias, median normalized to two-copies and then log _2_-transformed.

## Additional file

Additional file 1 Supplemental PDF file containing **Figures S1-S15**. (PDF 1540 kb)

## Abbreviations

AS: Assembly methods
CBS: Circular binary segmentation
CNAs: Copy number alterations
CNVs: Copy number variants
DOC: Depth of coverage
FP: False positive
FPR: False positive rate
GIAB: Genome in a bottle consortium
HMM: Hidden Markov models
RC: Read count
RP: Read pair
SGS: Second generation sequencing
SLM: Shifting level model
SR: Split read
SVs: Structural variations
TCGA: The Cancer Genome Atlas
TGS: Third generation sequencing
TP: True positive
TPR: True positive rate
WGS: Whole genome sequencing
