# Supplementary material for: XCAVATOR: accurate detection and genotyping of copy number variants from second and third generation whole-genome sequencing experiments
Source: BMC Genomics. 2017 Sep 21;18:747. doi: 10.1186/s12864-017-4137-0 (PMC5609061; doi:10.1186/s12864-017-4137-0)
Supplement: Additional file 1 — Supplemental PDF file containing Figures S1-S15. (PDF 1540 kb) [file 12864_2017_4137_MOESM1_ESM.pdf]

Supplemental Material to: "XCAVATOR: accurate detection and genotyping of copy number variants from second and third generation whole-genome sequencing experiments."

Alberto Magi[1], Tommaso Pippucci[2] Carlo Sidore[3]

[1]Department of Experimental and Clinical Medicine, University of Florence, Florence, Italy, [2]Medical Genetics Unit, Department of Medical and Surgical Sciences, Polyclinic Sant'Orsola-Malpighi, University of Bologna, Bologna, Italy., [3]Istituto di Ricerca Genetica e Biomedica, Consiglio Nazionale delle Ricerche (CNR), Monserrato, Cagliari, Italy.,

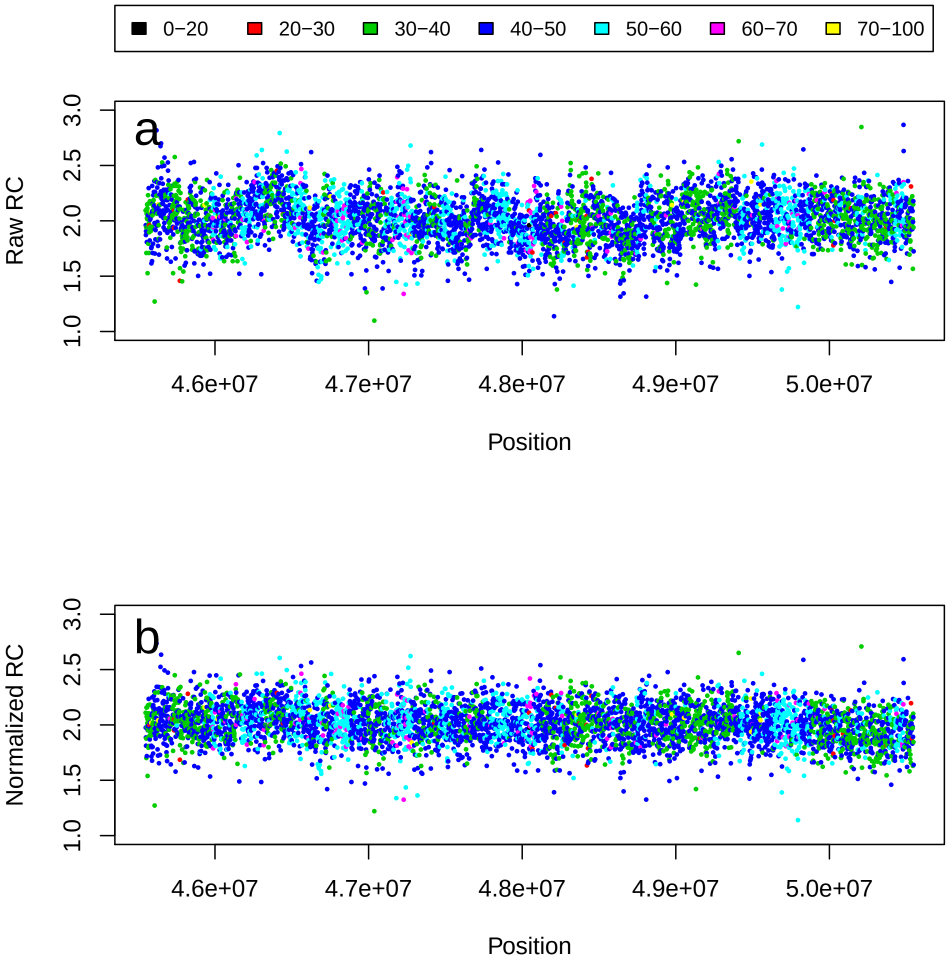

Figure 1: GC content and RC data. The two panels report the RC data as a function of GC-content % before (a) and after (b) median normalization. Panel b demonstrate that our median normalization scheme is capable to mitigate the effect of GC-content bias and generate a cleaner RC genomic profile. Each color in legend represents a bin of GC %. RC data were extracted from chromosome 1 of NA12878 sequenced at 50x with window size of 1000 bp.

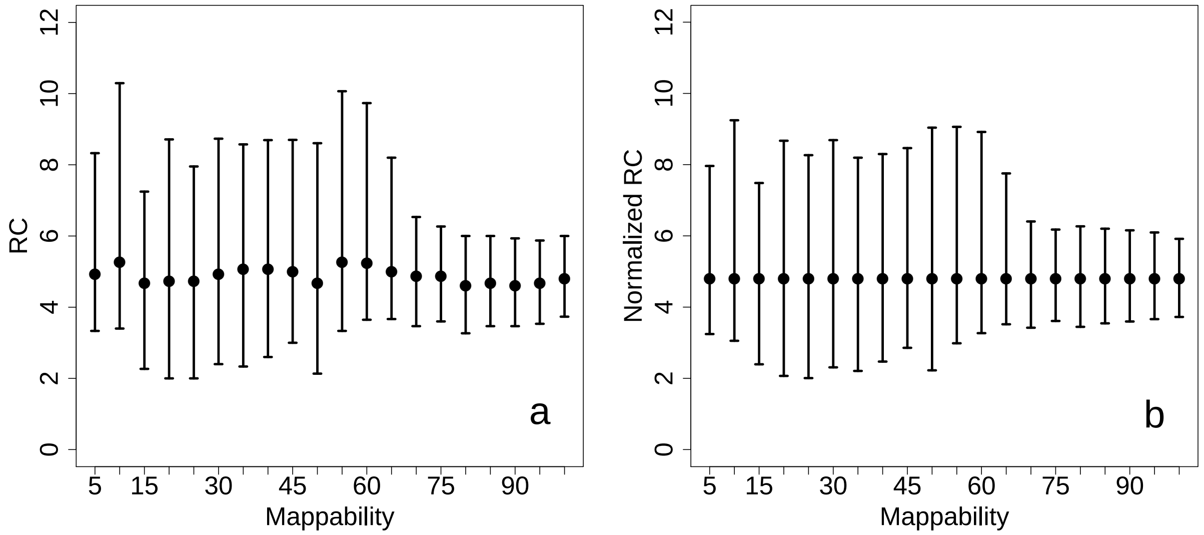

Figure 2: Mappability and RC data. The two panels report the RC data for low coverage WGS data as a function of mappability before (a) and after (b) median normalization. Panel b demonstrate that our median normalization scheme is capable to mitigate the effect of mappability. RC data were extracted from NA12878 downsampled at 5x with window size of 100 bp.

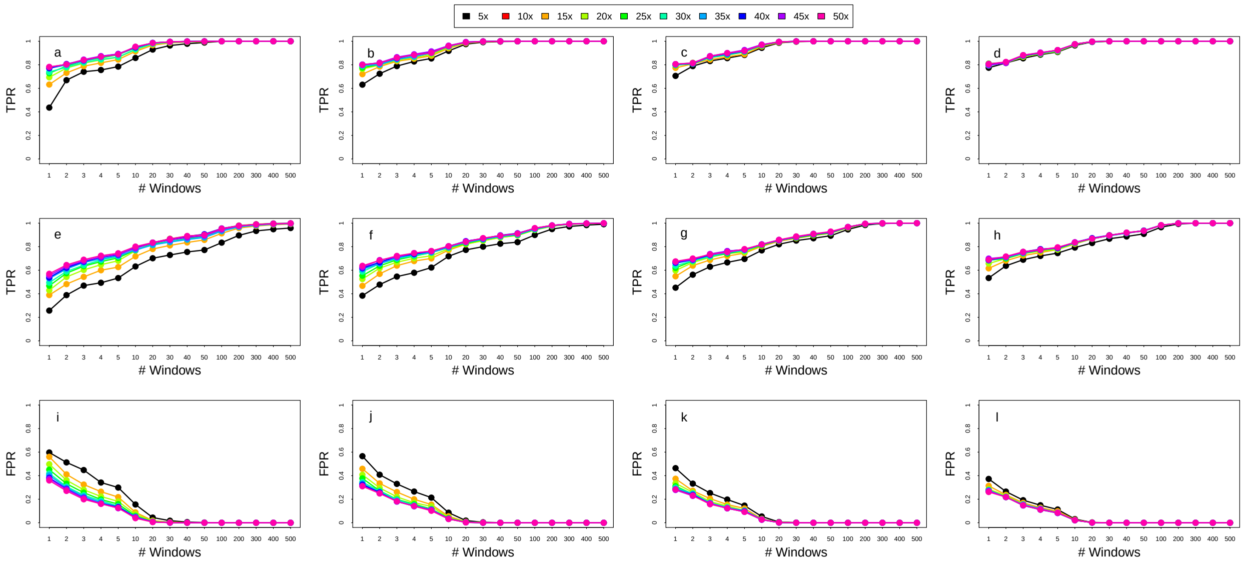

Figure 3: Prediction resolution of normalized RC data. The panels report the capability of N RC windows to predict deletions (a-d), duplications (e-h) and 2 copies regions (i-l) for different window sizes: 100 bp (a, e, i), 200 bp (b, f, j), 500 bp (c, g, k) and 1000 bp (d, h, l). The TPR of panels a-h are estimated as the proportion of simulated deletions (duplications) correctly predicted. The FPR of panels i-l are estimated as the proportion of 2 copies regions predicted as deletions and duplications. All the data reported in the panels are averaged across 1000 simulations.

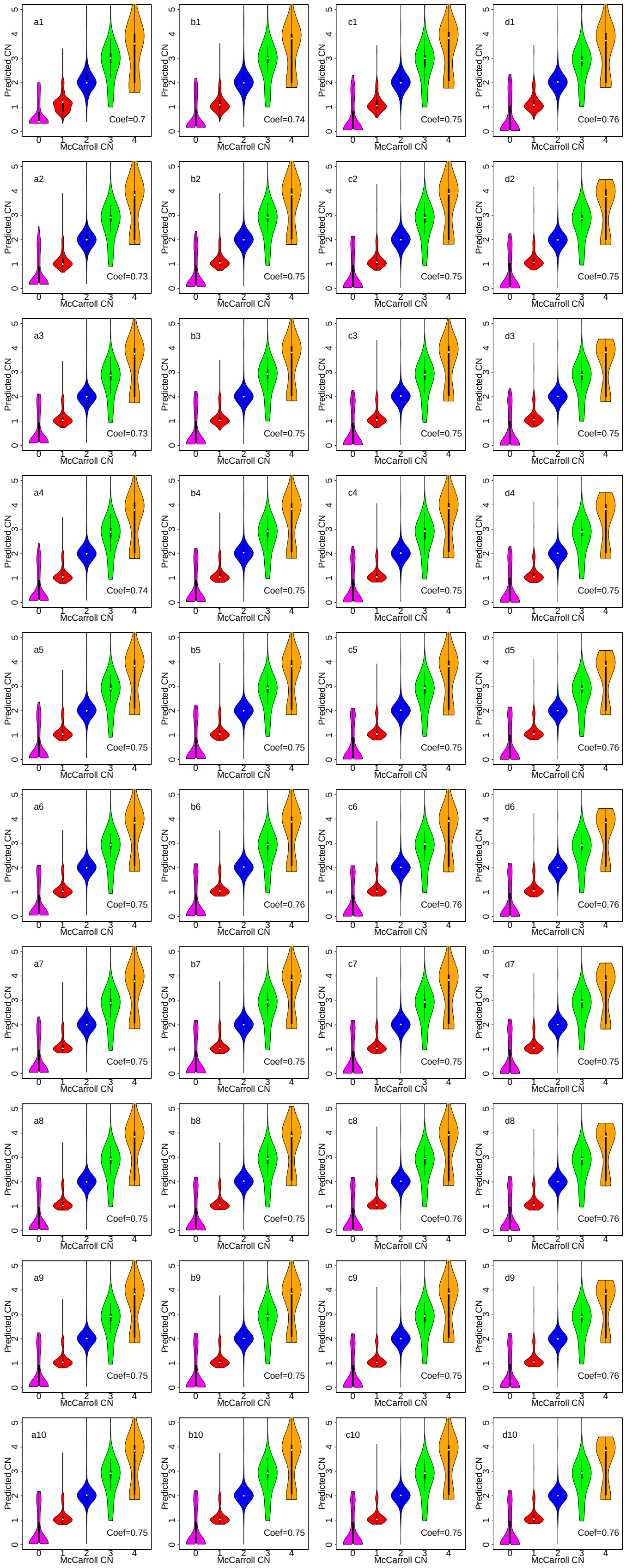

Figure 4: Correlation between normalized RC data and real copy number. The violin plot of the figure report the correlation between RC data and the absolute number of DNA copies previously predicted by McCarroll et al. for different sequencing coverages (a1-d1 for 5x, a2-d2 for 10x, a3-d3 for 15x, a4-d4 for 20x, a5-d5 for 25x, a6-d6 for 30x, a7-d7 for 35x, a8-d8 for 40x, a9-d9 for 45x, a10-d10 for 50x) and different window sizes (a for 100 bp, b, for 200 bp, c for 500 bp and d for 1000 bp).

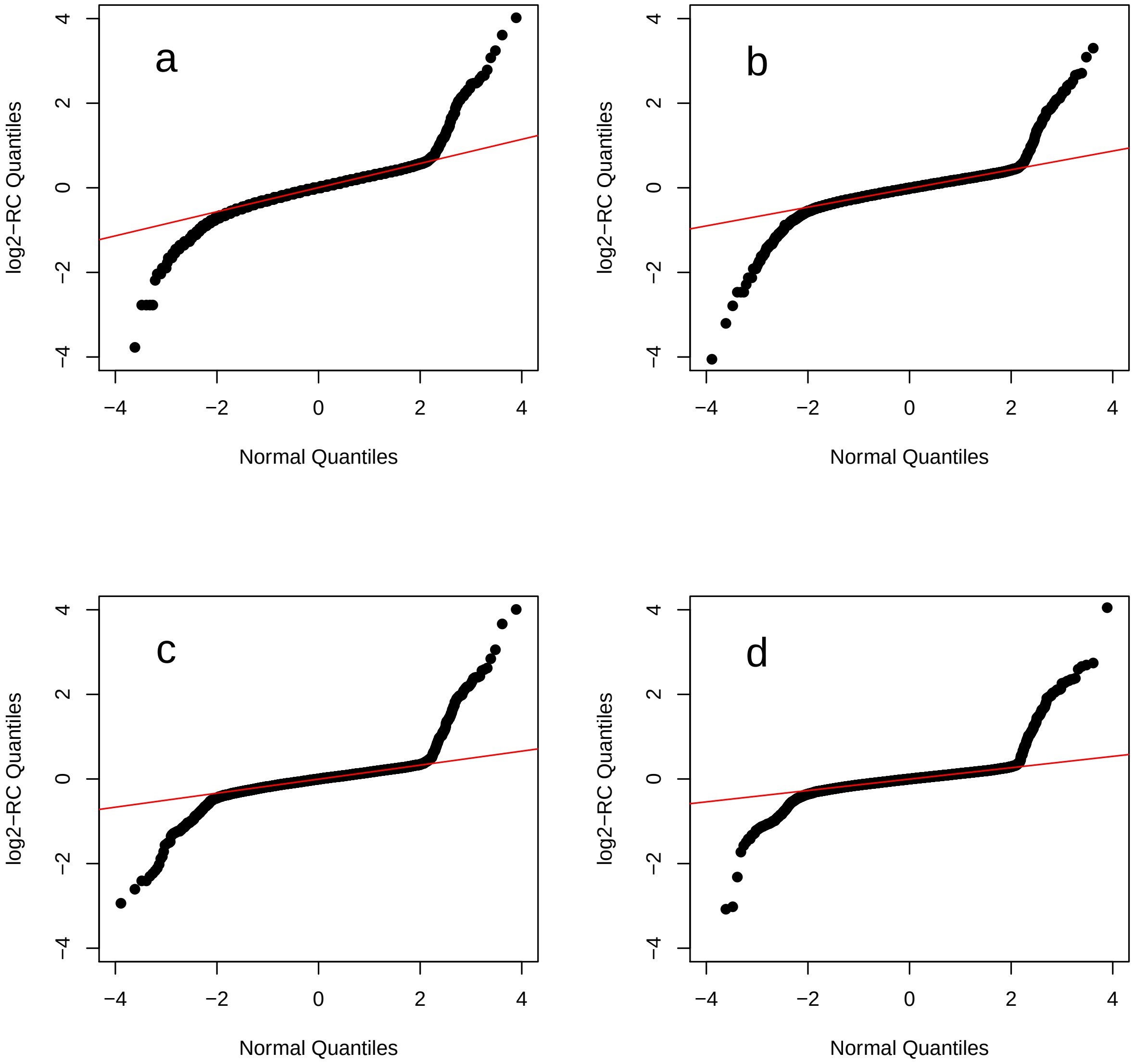

Figure 5: Normal QQ plot of log-transformed RC. We studied the distribution of $\log_2$-transformed RC for four different window sizes (a) 100 bp, (b) 200 bp, (c) 500 bp, (d) 1000 bp, and plotted their distributions relative to a standard normal distribution.

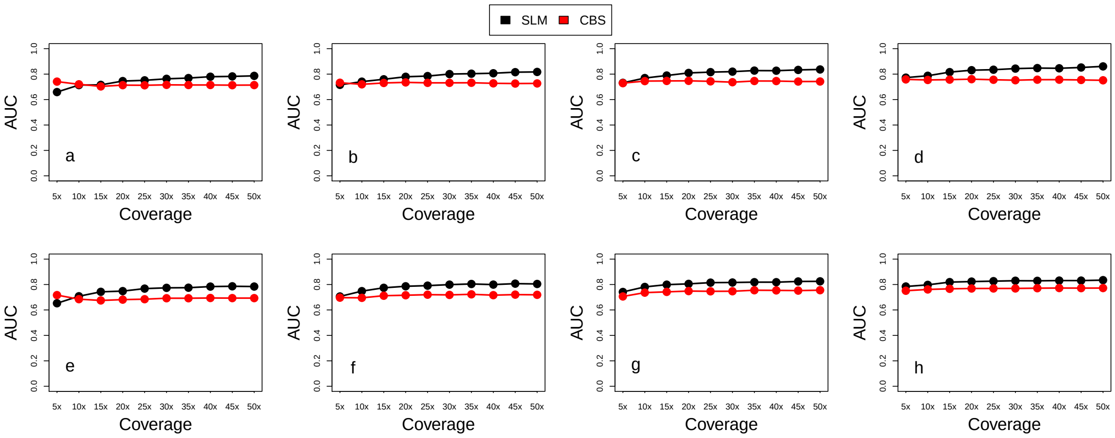

Figure 6: Area under the ROC curve of SLM and CBS. The panels report the area under the ROC curve of SLM and CBS algorithms as a function of sequencing coverages for different window sizes (a and e for 100 bp, b and f for 200 bp, c and g for 500 bp, d and h for 1000 bp). Panels a-d show the AUC for deletions, while panels e-h for duplications.

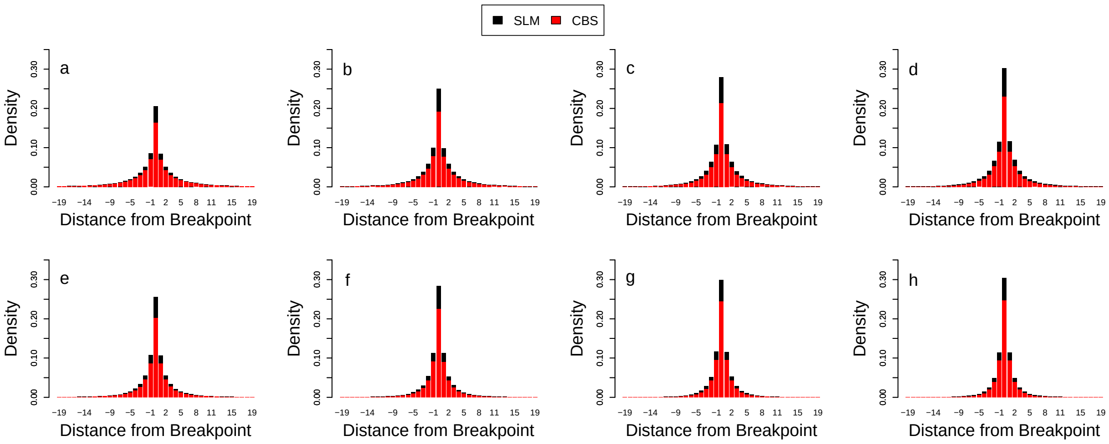

Figure 7: Breakpoints detection accuracy for SLM and CBS. Panels report breakpoint detection accuracy for deletions (a-d) and duplications (e-h) for different window sizes (a and e for 100 bp, b and f for 200 bp, c and g for 500 bp, d and h for 1000 bp). On the x axis is reported the distance between the predicted and the correct position. On the y axis is reported the fraction of breakpoints predicted at a given distance from the correct position.

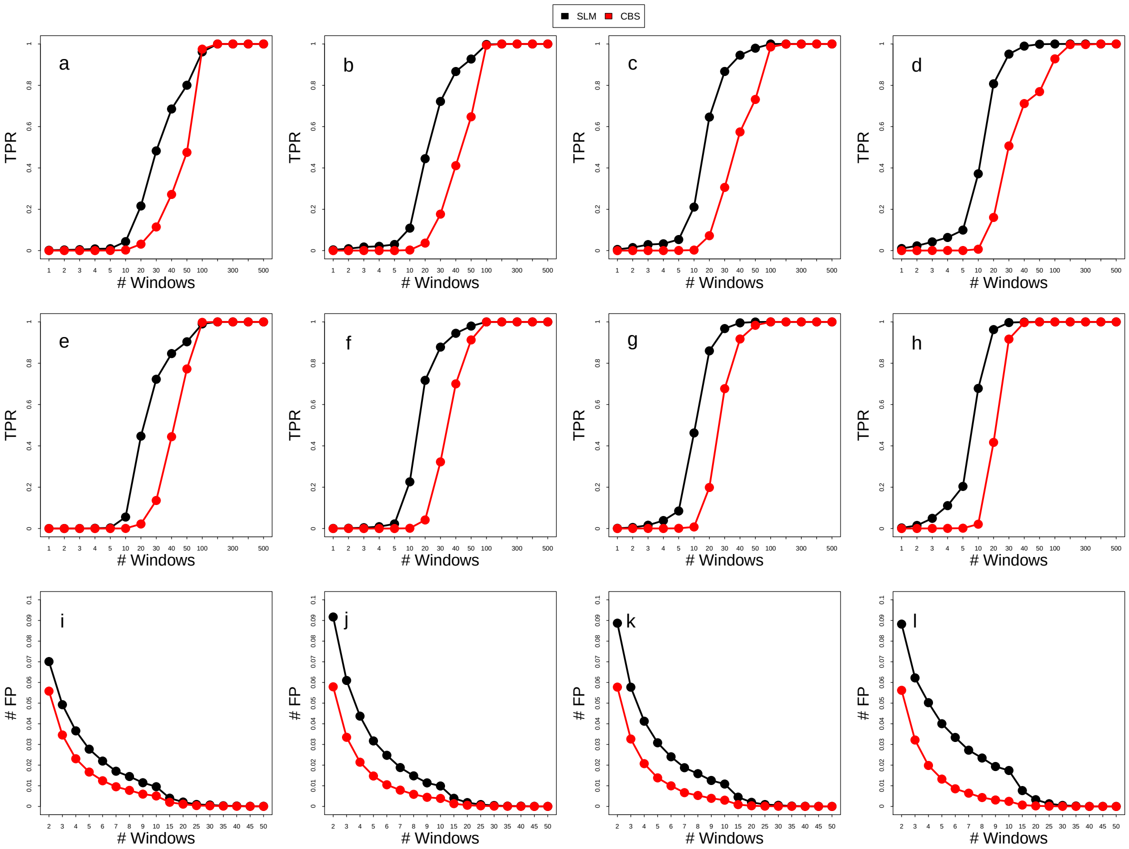

Figure 8: TPR and FP for SLM and CBS. Panels show the TPR (a-d for deletions, e-h for duplications) and # FP (i-l) for different window sizes: 100 bp (a, e, i), 200 bp (b, f, j), 500 bp (c, g, k) and 1000 bp (d, h, l). # FP is calculated as the average number of false positive events detected in all the synthetic chromosome we simulated.

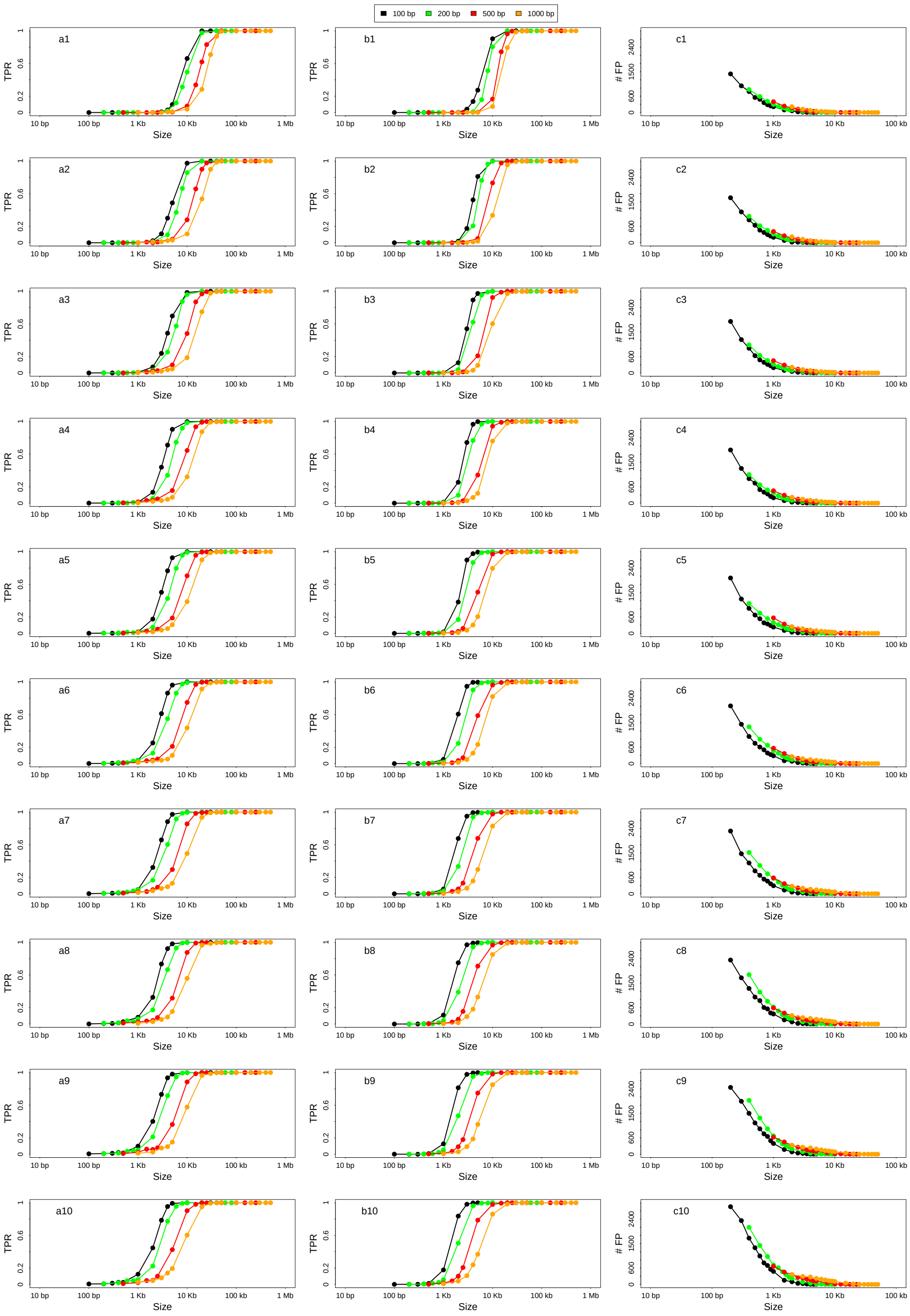

Figure 9: Resolution analysis for different sequencing coverages. Panels show the TPR (a1-a10 for deletions, b1-b10 for duplications) and FP (c1-c10) of SLM+FastCall to detect alterations of different size. TPR and FP are reported for different sequencing coverages (a1-c1 for 5x, a2-c2 for 10x, a3-c3 for 15x, a4-c4 for 20x, a5-c5 for 25x, a6-c6 for 30x, a7-c7 for 35x, a8-c8 for 40x, a9-c9 for 45x, a10-c10 for 50x). # FP is estimated as the total number of false positive events we can expect from the analysis of an entire human genome.

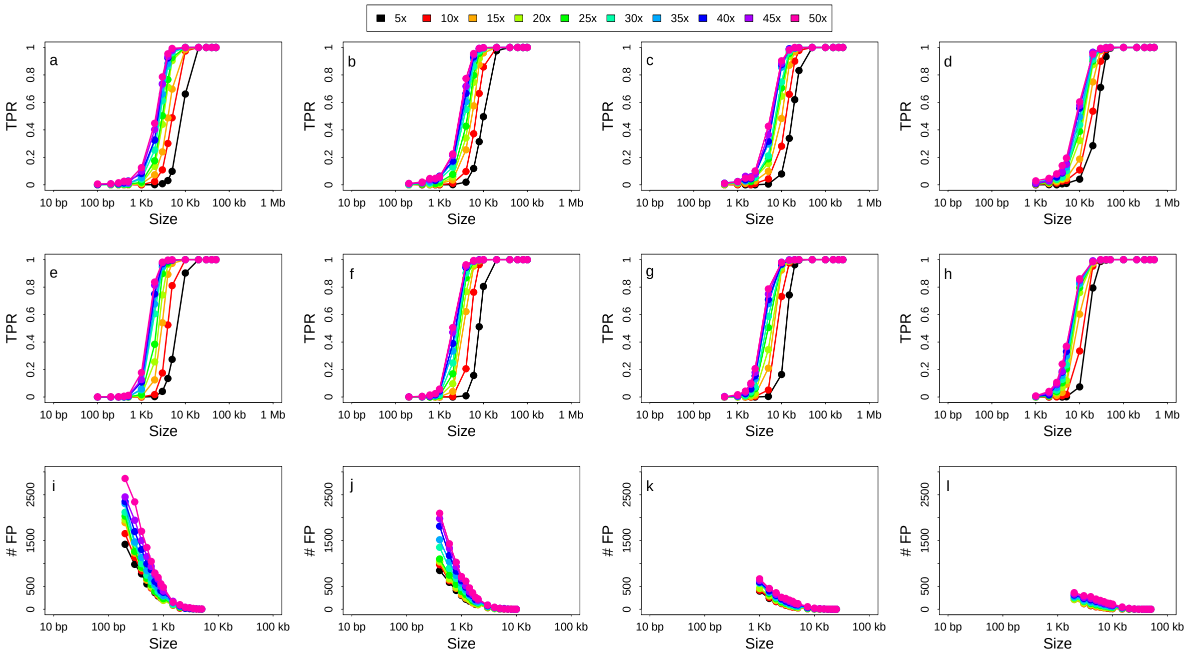

Figure 10: Resolution analysis for different window sizes. Panels show the TPR (a-d for deletions, e-h for duplications) and FP (i-l) of SLM+FastCall to detect alterations of different size. TPR and # FP are reported different window sizes: 100 bp (a, e, i), 200 bp (b, f, j), 500 bp (c, g, k) and 1000 bp (d, h, l). # FP is estimated as the total number of false positive events we can expect from the analysis of an entire human genome.

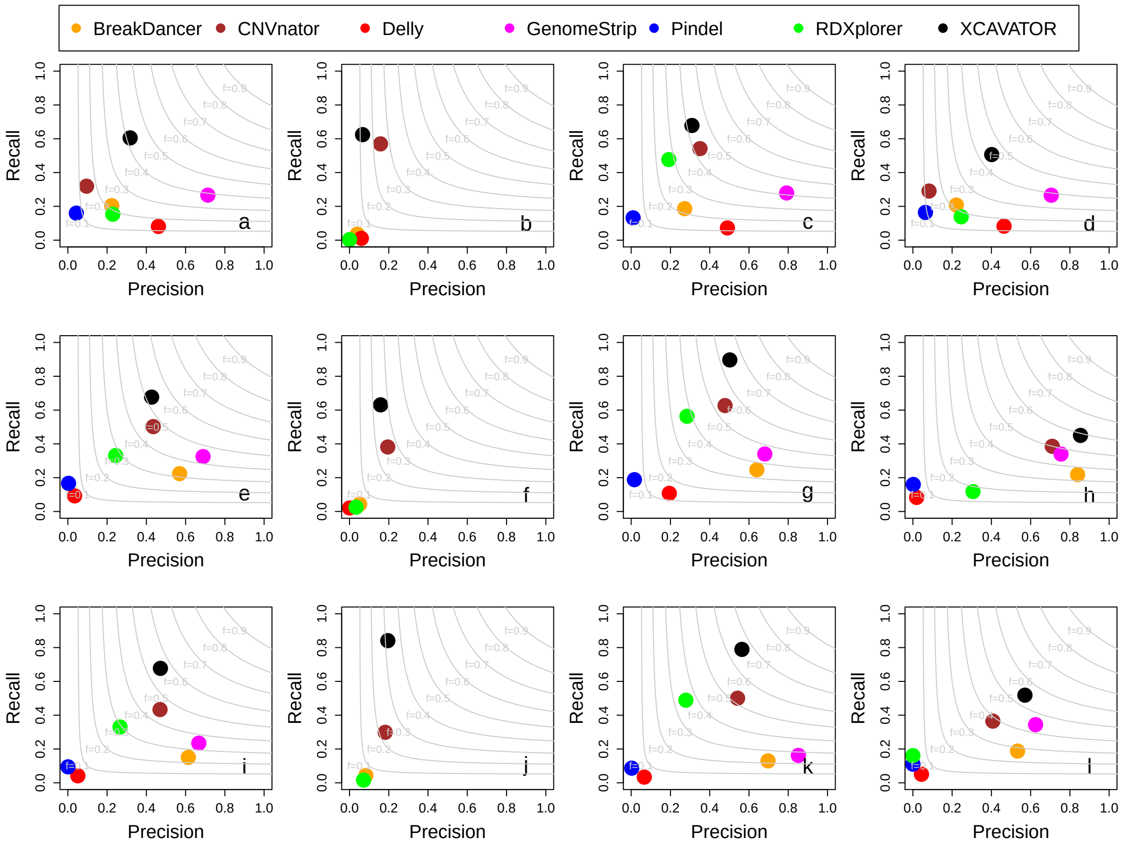

Figure 11: Precision and recall for the seven germline CNVs detection methods. Panels of the figure report precision and recall obtained by the seven tools in detecting CNVs of different size previously identified by 1KG pilot 1 (a-d), HapMap (e-h) and McCarroll (i-l). Panels a, e and i show precision and recall for all CNVs. Panels b, f and j show precision and recall for small ($size \leq 20Kb$) CNVs. Panels b, f and j show precision and recall for large ($size > 100kb$) CNVs. Panels c, g and k show precision and recall for medium ($20kb < size \leq 100kb$) CNVs. Panels d, h and l show precision and recall for small ($size \leq 20Kb$) CNVs. Light grey curves represent F-measure levels (harmonic mean of precision and recall).

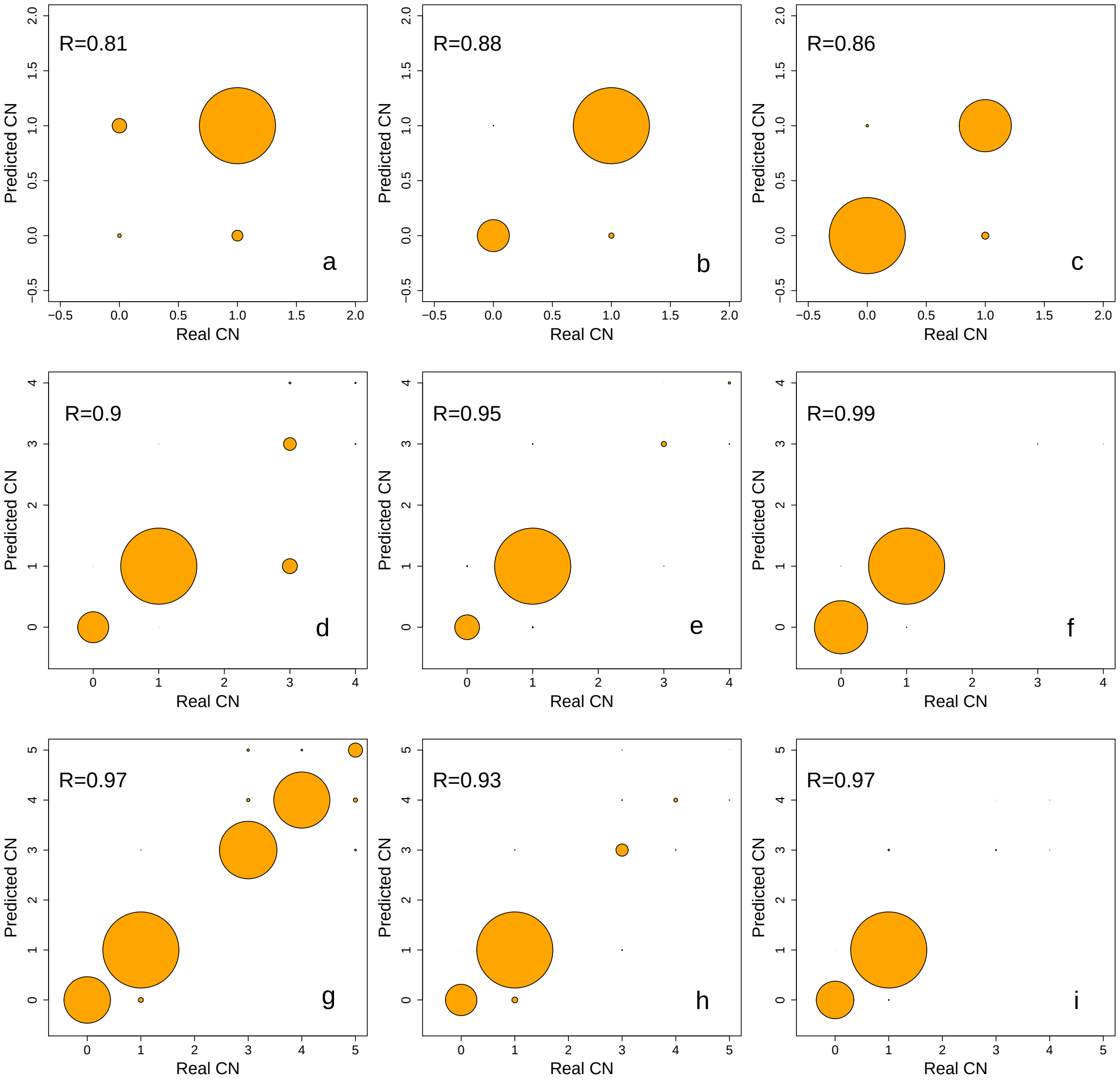

Figure 12: Absolute number of DNA copies inferred by FastCall for 1000 GP dataset. Panels report the correlation between the absolute number of DNA copies inferred by SLM+FastCall and those previously estimated by 1KG pilot 1 (a-c), HapMap (d-f) and McCarroll (g-i) for small ($size \leq 20Kb$, panels a, d and g), medium ($20kb \leq size \leq 100kb$, panels b, e and h) and large ($size \geq 100kb$, panels c, f and i) events. R is the Pearson correlation coefficient.

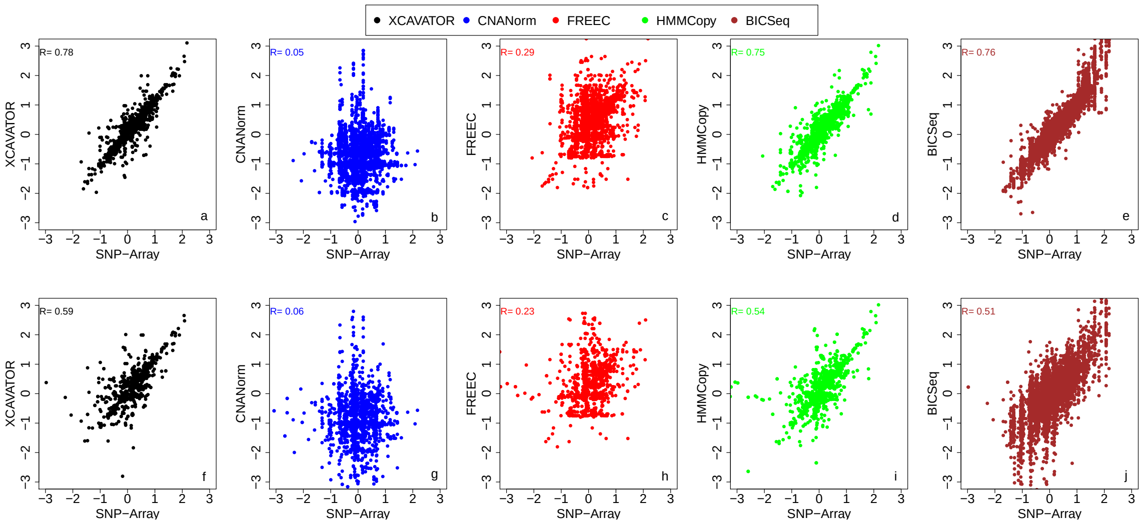

Figure 13: Log2-ratio median values correlation. Panels report the correlation between log2-ratio median values obtained from WGS and SNP-array for genomic segments detected by XCAVATOR (a, f), CNANorm (b, g), FREEC (c, h), HMMCopy (d, i) and BICSeq (e, j). Correlation analysis was performed for small ($\leq 20kb$, a-e) and large ($\geq 20kb$, f-j). R is the Pearson correlation coefficient.

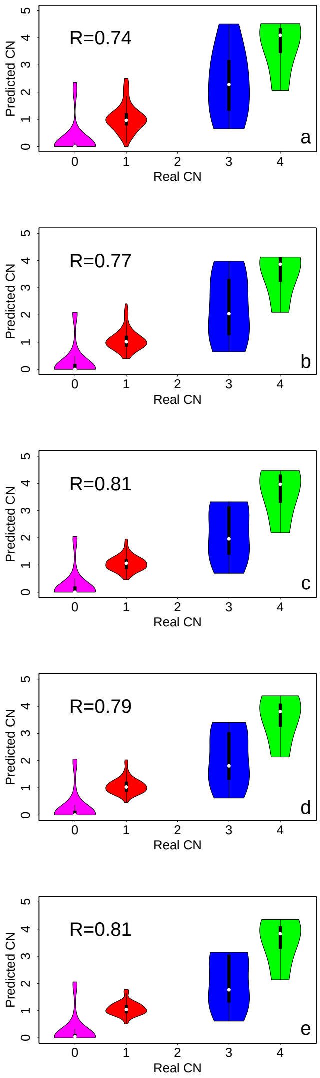

Figure 14: Absolute number of DNA copies inferred by FastCall for PacBio data. The violin plot of panels show the correlation between the absolute number of DNA copies obtained by DOC signals and those previously estimated by McCarroll et al. The correlation analysis has been performed for different sequencing coverages: 5x (a), 10x (b), 20x (c), 30x (d) and 45x (e). R is the Pearson correlation coefficient.

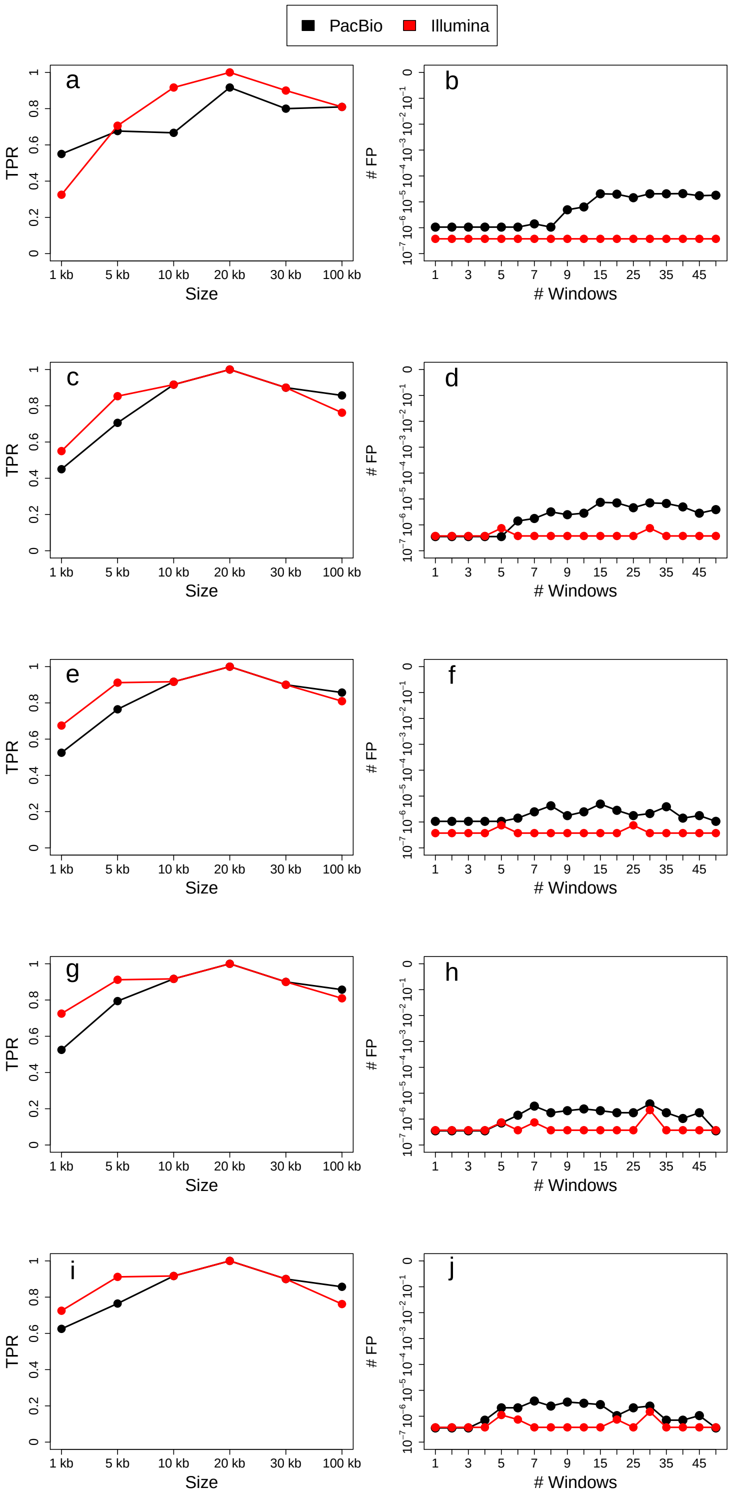

Figure 15: TPR and FPR of XCAVATOR on PacBio data. Panels summarize the TPR and FP frequency of CNVs detected by XCAVATOR with PacBio and Illumina data for different sequencing coverages: 5x (a-b), 10x (c-d), 20x (e-f), 30x (g-h) and 45x (i-j).
